# Isolation and characterization of a promoter responsive to salt, osmotic and dehydration stresses in soybean

**DOI:** 10.1590/1678-4685-GMB-2016-0052

**Published:** 2017-03-27

**Authors:** Alessandra Jordano Conforte, Fábia Guimarães-Dias, Anna Cristina Neves-Borges, Marta Bencke-Malato, Durvalina Felix-Whipps, Márcio Alves-Ferreira

**Affiliations:** 1 Department of Genetics. Universidade Federal do Rio de Janeiro (UFRJ), Rio de Janeiro, RJ, Brazil.; 2 Department of Botany. Universidade Federal do Estado do Rio de Janeiro (UNIRIO), Rio de Janeiro, RJ, Brazil.

**Keywords:** α-galactosidase gene, salt stress, drought-tolerant soybean, *cis*-acting element, raffinose degradation pathway

## Abstract

Drought stress is the main limiting factor of soybean yield. Currently, genetic engineering has been one important tool in the development of drought-tolerant cultivars. A widely used strategy is the fusion of genes that confer tolerance under the control of the *CaMV35S* constitutive promoter; however, stress-responsive promoters would constitute the best alternative to the generation of drought-tolerant crops. We characterized the promoter of α-galactosidase soybean (*GlymaGAL*) gene that was previously identified as highly up-regulated by drought stress. The β-glucuronidase (*GUS*) activity of Arabidopsis transgenic plants bearing 1000- and 2000-bp fragments of the *GlymaGAL* promoter fused to the *uidA* gene was evaluated under air-dried, polyethylene glycol (PEG) and salt stress treatments. After 24 h of air-dried and PEG treatments, the *pGAL*-2kb led to an increase in *GUS* expression in leaf and root samples when compared to the control samples. These results were corroborated by qPCR expression analysis of the *uidA* gene. The *pGAL*-1kb showed no difference in *GUS* activity between control and treated samples. The *pGAL*-2kb promoter was evaluated in transgenic soybean roots, leading to an increase in *EGFP* expression under air-dried treatment. Our data indicates that *pGAL*-2kb could be a useful tool in developing drought-tolerant cultivars by driving gene expression.

## Introduction

Soybean (*Glycine max L. Merr*) is a valuable commodity due to its utilization in the pharmaceutical industry, biodiesel production and food for humans and animals (Tran and Mochida, 2010). Therefore, the global soybean production was estimated to be 324.2 million metric tons in 2016/2017 (USDA, 2016), of which approximately 64% of its production is concentrated in the USA and Brazil. Despite this, the yield and production of soybean has been impacted by the occurrence of drought-stress. Recently, in 2012, the drought in the USA was the most intense since the 1950s, triggering a 20% loss of yield (Zulauf, 2012; Rippey, 2015). In the same period, the loss of Brazilian soybean production was approximately 13% due to drought stress (Conab, 2012). Moreover, the most severe drought stress period in Brazil occurred between 2003 and 2005, when the loss of soybean was greater than 20% of production (Polizel *et al.*, 2011). Therefore, the development of drought-tolerant soybean cultivars is crucial and should have high priority. Among the strategies available, the development of transgenic crops by the overexpression of drought-tolerant genes has been proven to be successful and can have a significant impact on agricultural production (Silvente *et al.*, 2012; Rahman *et al.*, 2016).

The constitutive cauliflower mosaic virus 35S RNA promoter (*CaMV35S*) has been broadly used to control the expression of several genes responsive to drought due to the strong and conspicuous activity presented when introduced in plant genomes (Luo *et al.*, 2013; Novák *et al.*, 2013; Bhauso *et al.*, 2014; Withanage *et al.*, 2015; Du *et al.*, 2016). However, the constitutive overexpression of genes can affect plant development and metabolism (Hsieh *et al.*, 2002; Homrich *et al.*, 2012). Indeed, the control of the expression of drought-tolerant genes by stress-responsive and/or tissue-specific promoters has been used as alternative for the elimination of negative effect of constitutive gene expression driven by the *CaMV35S* promoter (Chan Ju *et al.*, 2012; Banerjee *et al.*, 2013; Yan *et al.*, 2015). Recently, stress-responsive promoters, such as the *PeNAC1* (Wang *et al.*, 2016), *ZmGAPP* (Hou *et al.*, 2016), *BBX24* (Imtiaz *et al.*, 2015), and *GmNCED*1 and *GmMYB363P* promoters (Li *et al.*, 2014; Zhang *et al.*, 2014), have been characterized. Among gene promoters previously characterized as drought-responsive, the *AtRD29A* promoter showed stronger activity during water deficit stress in transgenic plants when compared to the control plants (Yamaguchi-Shinozaki and Shinozaki, 1993). Therefore, it has been successfully used to drive the expression of drought-resistant genes in different plant species (Kasuga *et al.*, 1999; Polizel *et al.*, 2011; Datta *et al.*, 2012; Saint Pierre *et al.*, 2012; Bihmidine *et al.*, 2013; Engels *et al.*, 2013). However, the use of the *AtRD29A* promoter is restricted by patent protection (Shinozaki *et al.*, 2007). Moreover, soybean promoters could be an alternative to avoid the use of exotic DNA in soybean transgenic plants. Therefore, the isolation and characterization of new drought-responsive promoters from soybean are crucial and will augment the set of tools available for the development of drought-tolerant cultivars.

In previous studies, we identified a soybean drought stress-responsive gene, the α-galactosidase gene (*GlymaGAL*) and characterized its expression pattern in sensitive (BR 16) and tolerant (EMBRAPA 48) soybean cultivars under drought stress conditions in the soil or hydroponic systems. *GlymaGAL* showed high expression levels, especially in the leaves of a drought-tolerant cultivar (EMBRAPA48) submitted to water deprivation (Guimarães-Dias *et al.*, 2012; Guimaraes-Dias F, 2013, PhD Thesis, Federal University of Rio de Janeiro, Rio de Janeiro). *At3g57520* (*AtSIP2*), the putative Arabidopsis homolog of *GlymaGAL*, was originally annotated as raffinose synthase (Taji *et al.*, 2002). However, recently, *AtSIP2* was characterized as an alkaline α-galactosidase belonging to the raffinose degradation pathway (Peters *et al.*, 2010). Studies have shown that the raffinose oligosaccharide family is present in large quantities in legumes and is involved in seed desiccation, cold and drought responses (Sengupta *et al.*, 2015). In our current study, we isolated and characterized *in silico* the 1004-bp (*pGAL*-1kb) and 2010-bp (*pGAL*-2kb) sequences upstream to the start codon of the *GlymaGAL*. Moreover, we evaluated their activities in the leaves and roots of Arabidopsis transgenic plants and soybean transgenic roots under drought and osmotic stress.

## Material and Methods

### *Cis-*element analyses

The genomic sequences of *pGAL*-1kb and *pGAL*-2kb, which are upstream of the start codon of the soybean α-galactosidase gene of the soybean genome (*GlymaGAL*), were obtained using the genome browser tool in the Phytozome database v.9 (Goodstein *et al.*, 2012). The *cis*-regulatory elements related to the response to water deficit were selected based on information from the literature (Urao *et al.*, 1993; Baker *et al.*, 1994; Yamaguchi-Shinozaki and Shinozaki, 1994; Iwasaki *et al.*, 1995; Abe *et al.*, 1997; Busk and Pagès, 1998; Abe *et al.*, 2003; Dubouzet *et al.*, 2003; Narusaka *et al.*, 2003; Tran *et al.*, 2004; Behnam *et al.*, 2013) (Table 1). The presence and frequency of these *cis*-elements in the *pGAL*-1kb and *pGAL*-2kb genes were determined by the PLACE database (Plant Cis Program-acting Regulatory) (Higo *et al.*, 1999).

**Table 1:** *Cis*-elements responsive to drought stress in the *pGAL* soybean promoter.

*Cis*-regulatory element^a^	Core sequence	Number of *Cis*-elements				Description
		1.0Kb	2.0Kb			
		(+) Strand	(-) Strand	(+) Strand	(-) Strand	
**DRE**	ACCGAC	0	0	1	0	Dehydration, high salinity and cold responsive
**GBOX**	CACGTG	0	0	1	0	Dehydration, high salinity, ABA and cold responsive
**MYCATERD1**	CATGTG	0	0	1	0	Dehydration responsive
**ABRE**	ACGTGTC	0	0	1	0	Dehydration, high salinity and low temperature responsive
**LTRECORE**	CCGAC	0	1	1	0	Low temperature, drought response
**MYBCORE**	CNGTTR	2	0	0	0	High salinity, ABA, heat, cold and dehydration responsive
**ACGTATERD1**	ACGT	3	3	4	4	Dehydration responsive
**MYCATRD22**	CACATG	1	0	1	1	Dehydration and ABA responsive
**MYCCONSENSUAT (CNNTG)**	CACATG/CACGTG/ CAGATG/CATGTG	2	2	2	2	Drought stress response
**ABRELATERD1**	ACGTG	2	0	2	2	Dehydration responsive
**MYB1AT**						
**(WAACCA)**	AAACCA/TAACCA/TGGTTA	1	1	1	0	Dehydration and ABA responsive
**MYB2 (YAACKG)**	CCGTTA	0	1	0	0	Dehydration responsive
**ABREATRD22**	RYACGTGGYR	0	0	0	0	ABA responsive

a The symbol W was used to represent A or T; the symbol R was used to represent A or G; the symbol Y was used to represent C or T; the symbol K was used to represent T or A; the symbol N was used to represent A or C or G or T.

### Isolation and cloning of the *GlymaGAL* promoters of soybean

The promoter sequences of the *GlymaGAL, pGAL*-1kb and *pGAL*-2kb genes were amplified using specific primers (Table S1). The genomic DNA used as a template was extracted from a drought-tolerant soybean cultivar (EMBRAPA48) according to a CTAB-based protocol (Doyle and Doyle, 1987). The amplification reactions were performed in a 50 μL final volume, which contained 100 ng of template DNA, 0.3 μM of each primer, 2 mM of MgSO4, 0.3 mM of each dNTP, 1X *Pfx* amplification buffer and 1 U of high fidelity platinum *Pfx* DNA polymerase (Thermo Fisher Scientific, Carlsbad, CA, EUA) according to the manufacturer's instructions. The reaction mixtures were submitted to the following cycling steps: 94 °C for 5 min, followed by 30 cycles of denaturation at 94 °C for 30 s, annealing at 50 °C or 55 °C (for *pGAL*-1kb and *pGAL*-2kb, respectively) for 1 min and extension at 68 °C for 2 min. The thermal profile ended with a final extension at 68 °C for 5 min. The PCR products were analyzed by 1% agarose gel electrophoresis and visualized by UV fluorescence after staining with ethidium bromide. Next, the PCR products were purified using a DNA Clean and Concentrator kit (Zymo Research CA, USA) according to the manufacturer's instructions. The concentration and purity analyses of each purified DNA were evaluated by a NanoDrop™ spectrophotometer ND-1000 (Thermo Fisher Scientific, Wilmington, DE, USA).

The purified products were first cloned into a gateway pENTR/D-TOPO entry vector (Life Technologies, Victoria, Australia) and used in the transformation of TOP10 chemically competent *Escherichia coli* cells according to the manufacturer's instructions, generating pGAL-1kb::pENTR and pGAL-2kb::pENTR. The plasmids of the positive colonies were extracted by a GeneJet Plasmid Miniprep kit (Fermentas, Glen Burnie, MD, USA) according to the manufacturer's instructions, and the concentration and purity of each purified DNA were analyzed by a spectrophotometer. At least three clones were sequenced, and the sequence was compared with the expected promoter sequence in the soybean reference genome (Goodstein *et al.*, 2012).

The pGAL-1kb::pENTR and pGAL-2kb::pENTR constructs were subsequently cloned by recombination into the binary vector Gateway^®^ pKGWFS7 (Invitrogen, Carlsbad, CA), which contained the *uidA* (encoding the *GUS* α, β-glucuronidase) and the *EGFP* (encoding the enhanced green fluorescent) gene sequences, generating the pGAL-1kb::pKGWFS7 and pGAL-2kb::pKGWFS7 clones (with the *GUS* and *EGFP* genes driven by *pGAL*-1kb and *pGAL*-2kb promoters, respectively). The recombination reactions were performed in a final volume of 8 μL according to the manufacturer's instructions under the following conditions: 1 μL of LR Clonase II (Invitrogen), 150 ng of pGAL-1kb::pENTR or pGAL-2kb::pENTR entry clones, 150 ng of the destination vector pKGWFS7 and TE buffer (1 mM EDTA, 10 mM Tris-HCl). After incubating each mixture for 1 h at 25 °C, 1 μL of proteinase k solution (2 μg/μL) was added, followed by incubation for 10 min at 37 °C. Next, 2 μL of each reaction was used to transform TOP10 chemically competent *E. coli* cells. Then, positive clones of each construct were confirmed by colony PCR reactions (using promoter-specific forward primers and the *EGFP*-specific reverse primer) (Table S1). The plasmids of these positive colonies were extracted by the GeneJet Plasmid Miniprep kit (Fermentas) according to the manufacturer's instructions and sequencing (Macrogen, Gasan-dong, South Korea). The concentration and quality analyses of each purified DNA were evaluated by a spectrophotometer.

The *AtRD29A* (positive control) and *CaMV35S* (negative control) promoter sequences (Yamaguchi-Shinozaki and Shinozaki, 1993) were cloned using the same vectors and methods described above to the pGAL constructs, generating the RD29A::pKGWFS7 and 35S::pKGWFS7 clones, respectively (with *GUS* and *EGFP* coding sequences submitted to control of the *RD29A* and *35S* promoters, respectively). We also used the DR5::GUS construct as a negative control (with the *uidA* gene driven by the *DR5* promoter) (Chen *et al.*, 2013).

### Transformation and selection of transgenic plants

The pGAL-1kb::pKGWFS7, pGAL-2kb::pKGWFS7, pRD29A::pKGWFS7 and pDR5::GUS constructs were introduced into *Agrobacterium tumefaciens GV3101* by electroporation, which was subsequently transferred into *Arabidopsis thaliana* ecotype Columbia (wild-type) by the floral dip method (Clough and Bent, 1998). The respective transformed plants, pGAL-1kb::GUS, pGAL-2kb::GUS, pRD29A::GUS and pDR5::GUS were grown in a pot containing substrate, vermiculite and perlite (2: 1: 0.5) at a controlled temperature of 22 °C ± 2 under a 16-h light/8-h dark photoperiod with a light intensity of 100 μmol m^−2^.s ^−1^ and 60% relative humidity.

Transgenic seeds with a single T-DNA were selected by segregation rates on one-half MS medium (Murashige and Skoog, 1962) agar plates containing 50 μg/mL kanamycin and maintained under the same conditions as mentioned above. The T1, T2 and T3 plantlets were transferred to soil and maintained under the same conditions until the seeds were collected. Then, three T3 homozygous transgenic lines expressing each construct were employed for abiotic stress treatments.

The pGAL-2kb::pKGWFS7 and 35S::pKGWFS7 constructs were introduced into *Agrobacterium rhizogenes* K599 by electroporation, which was subsequently used to transform roots of the tolerant soybean cultivar (Embrapa 48) using the syringe method (Kuma *et al.*, 2015). After seven days of co-cultivation, the plants were transferred into a selective medium of 100 μg/mL of cefotaxime and 100 μg/mL of spectinomycin, maintained under a 16-h light/8-h dark photoperiod and cycled at 25 °C ± 2 for 10 days. Transformation was confirmed through a visual inspection of *EGFP* expression using a fluorescence stereomicroscope (Leica M205 FA). Non-fluorescent roots were not excised to avoid injury. Finally, three transgenic plants for each construct (pGAL-2kb::GUS and 35S::GUS) were employed for abiotic stress treatments.

### Abiotic stress treatments

The activities of the *pGAL*-2kb, *pGAL*-1kb, *pRD29A* and *pDR5* promoters in the transgenic Arabidopsis plants under water privation stress were evaluated before and after salt stress, air-dried and polyethylene glycol (PEG) assays. In all experiments, the seeds harvested from pGAL-1kb::GUS (lines L1, L2, L3), pGAL-2kb::GUS (lines L1, L2, L3), RD29::GUS and DR5::GUS transgenic plants were initially surface sterilized and maintained in 4 °C for 4 days to break dormancy. Then, approximately 100 seeds for each line/treatment were germinated on one half-strength MS medium 1.2% agar in plates (150 mm diameter), which were positioned vertically and cultivated until 15 days old. They were grown under a continuous light photoperiod with a light intensity of 100 μmol m^−2^ s ^−1^, cycled at 22 °C ± 2 and 60% relative humidity and then submitted to four different treatments.

In the drought treatment by air drying (air-dried), a set of plates harboring 20 seedlings of each Arabidopsis transgenic line remained open for 6, 12 or 24 h (Rodrigues *et al.*, 2012; Nobres *et al.*, 2016). Meanwhile, a set of plates with control plants remained closed. For the soybean transgenic roots submitted to the air-dried treatment, a set of Magenta vessel GA-7 (Sigma) harboring 15 soybean plants remained open for 24 h, while a set of Magenta vessels with control plants remained closed.

In the PEG treatment, 15 seedlings of each Arabidopsis transgenic line were transferred to plates containing PEG 8000 (Verslues *et al.*, 2006). In this case, after the seeds germinated on one half-strength MS medium agar until 15 days old, the seedlings were transferred to plates (150 mm diameter) with one half-strength MS medium agar supplement (700 g PEG 8000 diluted in 1 L of water), submitting the seedlings to a water potential (Ψ) = −1.7 MPa. Meanwhile, control plants were maintained in a PEG-free solution. The PEG-infused plates were incubated overnight (approximately for 15 hours) before the plants were transferred.

Subsequently, the salt-stress treatments with 20 mL of 200 mM NaCl were added to cultivation medium harboring 20 seedlings of each transgenic line (Soussi *et al.*, 1998), which were submitted to this condition for 6, 12 or 24 h. Control plants were maintained in a NaCl-free solution.

In all treatments, control plants were collected at the end of the experiment to rule out external changes or influences in the results. After the stress treatments, qualitative and quantitative *GUS* analyses were performed. All treatments were conducted with three independent biological replicates.

### Histochemical *GUS* assay of Arabidopsis transgenic plants

The histochemical *GUS* assay was performed after stress application in the transgenic seedlings for 6, 12 or 24 h in the air-dried treatment, PEG treatment or salt treatments. The histochemical *GUS* assay was performed following the methods of Jefferson (1989) to assess the promoter activity in roots and leaves of the treated seedlings. The seedlings were observed under a stereomicroscope (Leica S8 APO) with a magnification of 10X and photographed by a Leica EC3 camera, and the image was adjusted in high resolution using LASEZ software version 3.0 (Leica). The histochemical *GUS* assay was performed with three independent biological replicates and three plants for each line.

### Total RNA isolation and transcript level analysis

To validate the results obtained by histochemical *GUS* assay and to quantitatively analyze the *pGAL-*2kb activity, qPCR assays were performed using root and leaf RNA samples of three lineages (pGAL-2kb::GUS (L1, L2 and L3), pRD29A::GUS (positive control) and pDR5::GUS (negative control)) submitted to the air-dried, PEG or salt stress conditions at 24 h and the non-stressed condition (control plants). The leaf RNA samples of each line/treatment (a total of five plants for each line/treatment) were extracted with Trizol Reagent (Invitrogen), followed by TURBO DNase enzyme (Ambion, Thermo Fisher) treatment according to the manufacturer's instructions. Meanwhile, the root RNA samples each line/treatment (a total of 10 plants for each line/treatment) were extracted using the RNeasy Plant Mini Kit (Qiagen, Inc., Valencia, CA, USA) followed by DNase treatment, as indicated by the manufacturer's protocol. The total RNA concentration and purity were determined using a spectrophotometer, while the RNA integrity was tested by electrophoretic separation in a 1% agarose gel. Three independent biological samples were collected for the relative expression studies.

The cDNA synthesis reactions with leaf and root RNA samples were initially performed using the SuperScript III Reverse Transcriptase enzyme (Invitrogen) following the manufacturer's instructions. The resulting cDNAs were used for qPCR assays run in triplicate. For the qPCR reactions, the *uidA* (*GUS*) gene-specific primers were used (Table S1).

The qPCRs were carried out in optical 96-well plates in a 7500 Fast Real-Time PCR detection system (Applied Biosystems) following the manufacturer's instructions. The amplification reactions were performed in a 20 μL final volume containing 10 μL cDNA (1:50); *SYBER* ^®^ *Green* 1X (Thermo Fisher Scientific); 0.4 μM of each primer (forward and reverse) (Síntese biotecnologia); 0.025 mM dNTP; PCR buffer (-Mg) 1X; 3 mM MgCl_2_; 0.25 U Platinum *Taq* DNA Polymerase (Thermo Fisher Scientific) and 0.4 μL ROX reference dye (Thermo Fisher Scientific).

The reaction mixtures were incubated at 94 °C for 5 min, followed by 40 cycles of 94 °C for 15 s, 60 °C for 10 s, 72 °C for 15 s and 60 °C for 35 s. Subsequently, a melting curve analysis was run from 30 °C to 100 °C for 1 min.

The melting curve and gel electrophoresis analyses of the amplification products confirmed that the primers amplified only a single product of expected size (data not shown). The primer set efficiencies and the Ct cutoff cycles (cycle thresholds) were estimated for each experimental set by *Online* real-time PCR Miner software (Zhao and Fernald, 2005), and these values were converted into normalized relative quantities (NRQs) by the program QBASE version 1.3.5 (Hellemans *et al.*, 2007). To determine the most stable combination of the reference genes *At4g34270, At4g38070* and *At5g12240* (Czechowski *et al.*, 2005) we used NormFinder software (Andersen *et al.*, 2004). As the *At4g34270* and *At4g38070* genes exhibited a stable expression pattern under abiotic stress, they were used as housekeeping genes for the normalization of *uidA* expresion. The quantitative expression data were analyzed statically by Student's *t*-test and variance analysis (ANOVA) methods using Assistat v 7.7 software (Silva and Azevedo, 2002).

## Results

### *In silico* analysis of the frequency of water deficit response *cis*-elements

**Figure 1:**
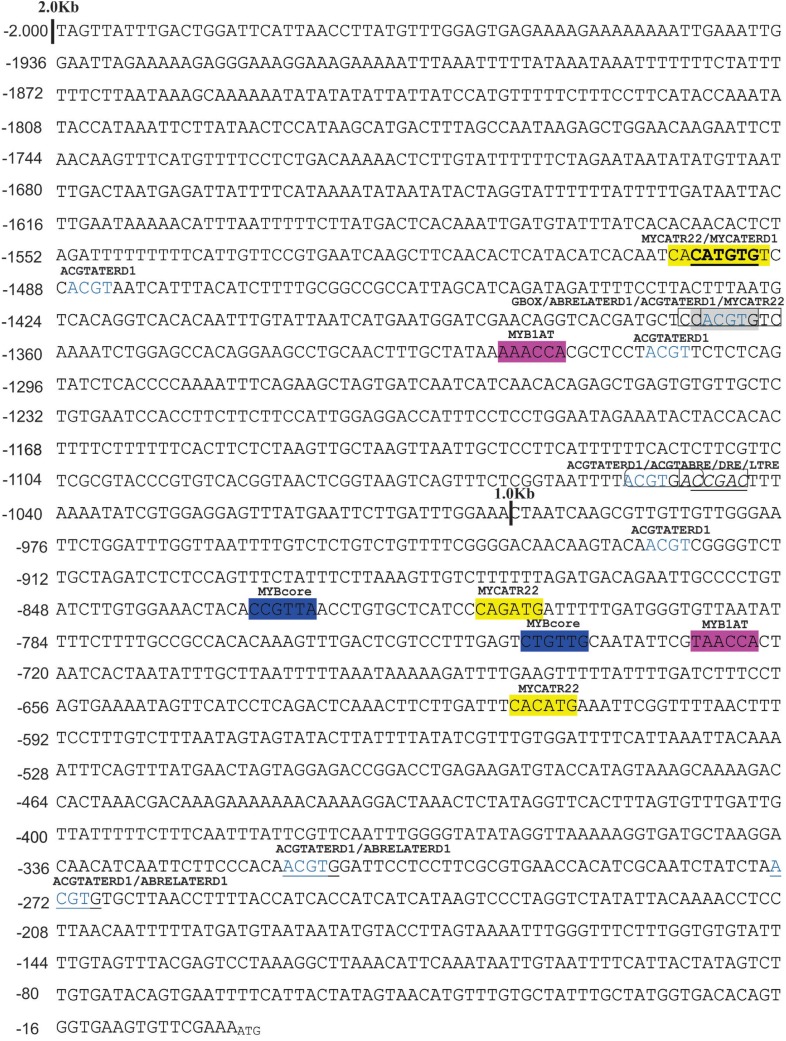
Genomic DNA sequence and different segments with different *cis*-elements in the drought-stress response of the promoter of the *GlymaGAL* gene from soybean. The immediate upstream nucleotide of the start codon of *GlymaGAL* is designated as position 1. The sequence represents a single-stranded DNA.

*In silico* analysis of the 2-kb fragment of the *GlymaGAL* promoter allowed us to identify 13 *cis-*acting elements previously associated with the water deficit response (Table 1) (Urao *et al.*, 1993; Baker *et al.*, 1994; Yamaguchi-Shinozaki and Shinozaki, 1994; Iwasaki *et al.*, 1995; Abe *et al.*, 1997; Busk and Pagès, 1998; Abe *et al.*, 2003; Dubouzet *et al.*, 2003; Narusaka *et al.*, 2003; Tran *et al.*, 2004; Behnam *et al.*, 2013). The presence and frequency of these *cis*-elements upstream of the start codon of *GlymaGAL* revealed that the *pGAL*-1kb and *pGAL*-2kb sequences have several putative water deficit response *cis*-acting elements (Figure 1). Among the 13 different *cis-*acting elements identified as associated with the drought response, only the ABREATRD22 motif was not found in the *GlymaGAL* promoter. However, the frequency of *cis*-elements was distinct over each promoter fragment (Table 1, Figure 1). The DRE, MYCATERD1, ABRE and GBOX motifs were found exclusively in *pGAL*-2kb (Table 1).

### Activity profile of the *pGAL*-1kb and *pGAL*-2kb promoters in transgenic Arabidopsis under water stress conditions

**Figure 2:**
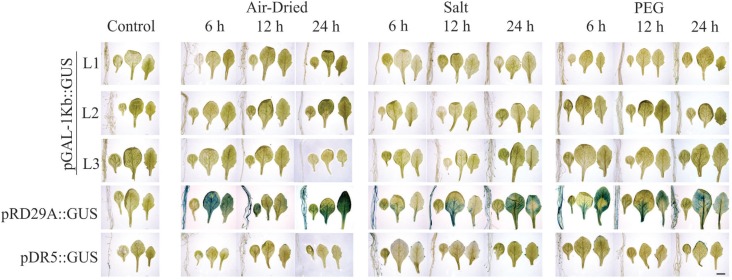
pGAL-1kb::GUS histochemical assay. Three transgenic lineages, pGAL-1kb::GUS (L1, L2 and L3), pRD29::GUS positive control and pDR5::GUS negative control, were submitted to control and 6, 12 and 24 h of the air-dried, salt stress and PEG conditions. The order of the sample photos is as follows: root, cotyledonal leaf, young leaf and totally expanded leaf. The data shown are representative of three independent lines (n = 3). Scale bars = 2 mm.

**Figure 3:**
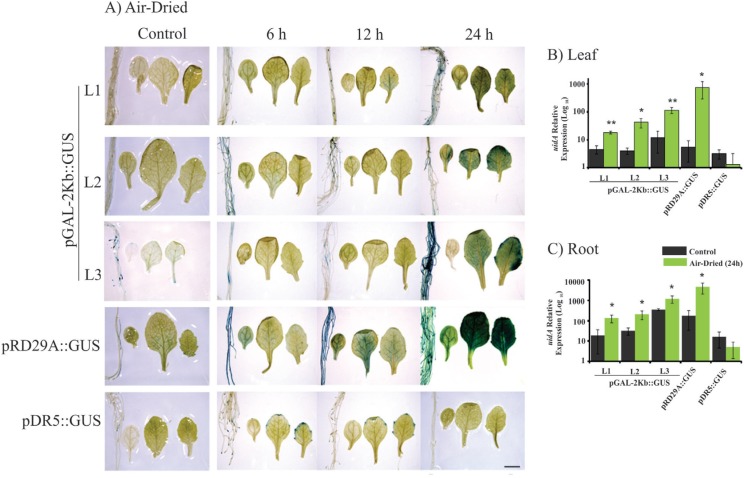
Histochemical analysis and expression profile analyses of the *GUS* reporter gene under control of the *pGAL*-2kb promoter. The order of the sample photos is as follows: root, cotyledonal leaf, young leaf and totally expanded leaf. A) The leaves and roots of plants grown under normal conditions and air-dried treatment (6, 12 and 24 h) were compared to the pRD29::GUS and pDR5::GUS plants under normal conditions and treated (6, 12 and 24 h). The data shown are representative of three independent lines (n = 3). B and C). The expression levels of *uidA* mRNA in leaves and roots under control (non-treated) and air-dried treatment. Values are means ± SD (n = 3). The relative expression values, represented on the y-axis, were obtained by qPCR experiments and calculated using the 2^−ΔΔCt^ method. The At4g34270 and At4g38070 genes were used as endogenous controls to normalize data. Asterisks indicate significant differences of samples under air-dried treatment and non-treatment (Student's *t*-test * p < 0.05 and ** p < 0.01). Total RNA was extracted from the leaves and roots of three independent T3 lines of 2-week-old transgenic plants after 24 h of drought (air-dried) treatment. Scale bars = 2 mm.

**Figure 4:**
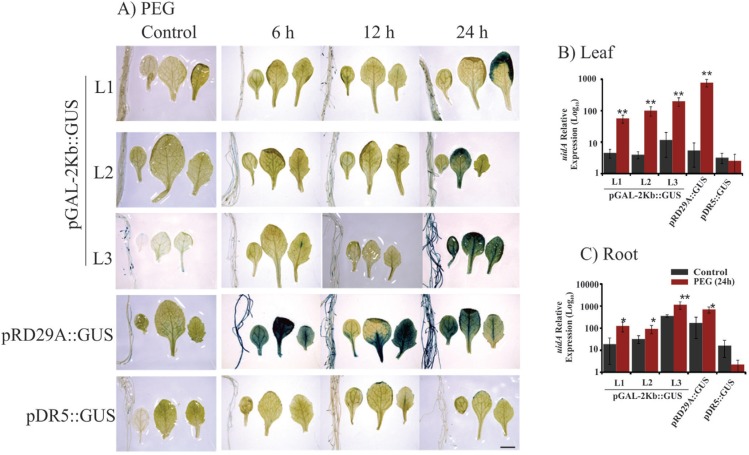
Histochemical analysis and expression profile analyses of the *GUS* reporter gene under control of the *pGAL*-2kb promoter. The order of the sample photos is as follows: root, cotyledonal leaf, young leaf and totally expanded leaf. A) The leaves and roots of plants grown under normal conditions and PEG treatment (6, 12 and 24 h) were compared to the pRD29::GUS and pDR5::GUS plants under normal conditions and treated conditions (6, 12 and 24 h). The data shown are representative of three independent lines (n = 3). B and C) The expression levels of *uidA* mRNA in leaves and roots under PEG treatment and no treatment. Values are means ± SD (n = 3). The relative expression values, represented on the y-axis, were obtained by qPCR experiments and calculated using the 2^−ΔΔCt^ method. The At4g34270 and At4g38070 genes were used as endogenous controls to normalize data. Asterisks indicate significant differences of samples under PEG treatment and no treatment (Student's *t*-test * p < 0.05 and ** p < 0.01). Total RNA was extracted from the leaves and roots of three independent T3 lines of 2-week-old transgenic plants after 24 h of PEG treatment. Scale bars = 2 mm.

**Figure 5:**
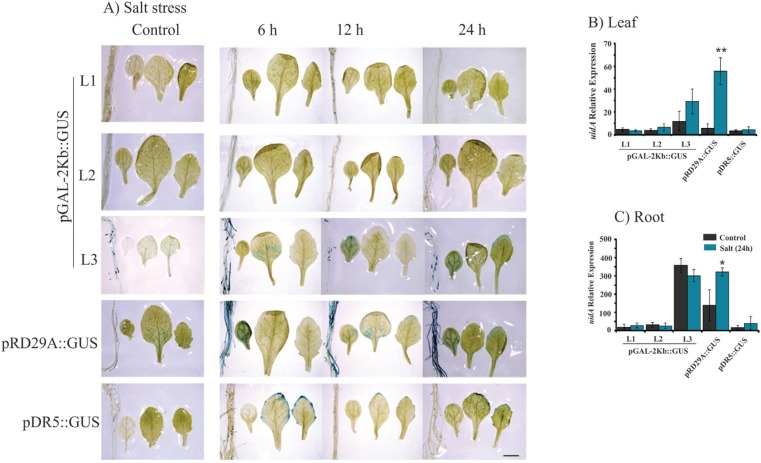
Histochemical analysis and expression profile analyses of the *GUS* reporter gene under control of the *pGAL*-2kb promoter. The order of the sample photos is as follows: root, cotyledonal leaf, young leaf and totally expanded leaf. A) The leaves and roots of plants grown under normal conditions and salt treatment (6, 12 and 24 h) were compared to the pRD29::GUS and pDR5::GUS plants under normal conditions and treated conditions (6, 12 and 24 h). The data shown are representative of three independent lines (n = 3). B and C) The expression levels of *uidA* mRNA in leaf and roots samples under control (non-treated) and salt treatment. Values are means ± SD (n = 3). The relative expression values, represented on the y-axis, were obtained by qPCR experiments and calculated using the 2^−ΔΔCt^ method. The At4g34270 and At4g38070 genes were used as endogenous controls to normalize data. Asterisks indicate significant differences of samples under salt treatment and no treatment (Student's *t*-test * p < 0.05 and ** p < 0.01). Total RNA was extracted from the leaves and roots of three independent T3 lines of 2-week-old transgenic plants after 24 h of salt treatment. Scale bars = 2 mm.

Three plants of each transgenic lineage were evaluated for the respective promoter expression level by histochemical *GUS* assay and photographed in bright field microscopy after 6, 12 and 24 h of the air-dried, PEG and salt stress treatments. Plants bearing the pRD29A::GUS and pDR5::GUS constructs were used as positive and negative controls, respectively (Figures 2-5).

Plants bearing the pGAL-1kb::GUS construct did not show any *GUS* activity when submitted to stress treatments (Figure 2). However, the pGAL-2kb::GUS transgenic plants submitted to the air-dried treatments for 24 h showed a strong *GUS* activity in roots and leaves. However, no signal was observed in these plants at earlier time points (6 and 12 h) (Figure 3A). PEG treatment of pGAL-2kb::GUS transgenic plants triggered the same temporal expression pattern as the air-dried treatment; *GUS* activity was observed after 24 h (Figure 4A). qPCR analysis of *pGAL*-2kb transgenic plants substantiated the histochemical assays, showing that the *uidA* gene was significantly up-regulated (Student's *t*-test * p < 0.05 and ** p < 0.01) in the leaves and roots of transgenic lines when submitted to the air-dried (Figure 3B, C) and PEG treatments after 24 h (Figure 4B, C). In contrast, no signal was observed in pGAL-2kb::GUS transgenic plants under salt stress in leaves and roots (Figure 5A). Again, this result was confirmed by qPCR (Figure 5B, C).

### Activity profile of the *pGAL*-2kb soybean promoter in soybean transgenic roots under drought stress conditions

**Figure 6:**
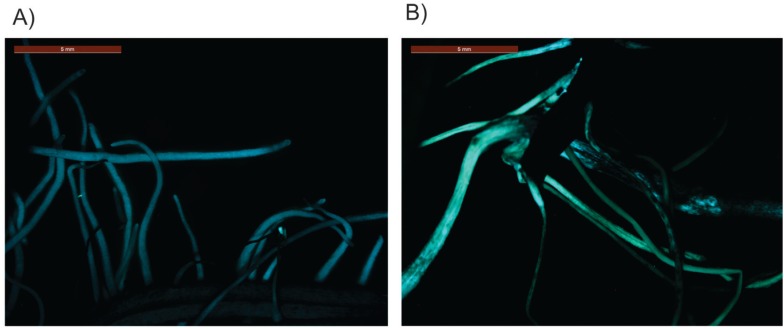
Expression *EGFP* in *A. rhizogenes* transformed soybean roots submitted to 24 h of air- dried treatment. (A) 35S::GUS (B) pGAL-2kb::GUS.

To investigate whether the *pGAL*::2kb promoter is also active in soybean plants, we performed a soybean root transformation according to Kuma *et al.* (2015). Transformed roots submitted to the air-dried treatment showed a substantial increase in the *EGFP* signal when compared to untransformed roots under the same stress condition (Figure 6).

## Discussion

In this study, we evaluated the activity of *pGAL*-1kb and *pGAL*-2kb promoter sequences of the soybean α-galactosidase gene in Arabidopsis and soybean transgenic plants under drought and salt stress. The *pGAL*-2kb promoter had high activity in the roots of Arabidopsis and soybean transgenic plants submitted to water deficit by the air-dried treatment (Figure 3 and 6). Similar results were also observed in Arabidopsis leaves submitted to the air-dried and PEG treatments (Figure 3 and 4). However, the *pGAL*-2kb promoter sequence did not show a significant change in the activity of Arabidopsis transgenic plants under salt stress conditions (Figure 5). These results showed that the *pGAL*-2kb promoter sequence is able to drive inducible expression primarily by drought stress.

Our results showed that the *pGAL*-1kb sequence lacks activity under the tested conditions, which is likely due to the absence of specific *cis*-elements involved in controlling the dehydration stress response (Figure 2). Other authors have also shown the importance of distal promoter regions in stress-associated responses (Behnam *et al.*, 2013; Imtiaz *et al.*, 2015). For example, the 3.0-kb *AtNCED3* promoter sequence was able to drive *uidA* expression in response to dehydration, while the 1.5-kb *AtNCED3* promoter region was not functional (Tan *et al.*, 2003; Behnam *et al.*, 2013). Imtiaz *et al.* (2015) showed that the 2.7-kb *CmBBX24* promoter sequence of Chrysanthemum has activity in Arabidopsis transgenic plants under drought and salt stress, but the deletion of fragments from the promoter's distal end led to a lower promoter activity under drought stress. When compared to the *pGAL*-1kb promoter sequence, *pGAL*-2kb has four additional *cis*-motifs previously associated with the response to drought stress, including one ABRE, one MYCATERD1, one GBOX and one DRE (Table 1). Previous results have shown that the presence of these *cis*-elements has been associated with the response to dehydration and/or abiotic stresses (Abe *et al.*, 1997; Narusaka *et al.*, 2003; Lata and Prasad, 2011; Behnam *et al.*, 2013). However, only experimental data can validate the role of these *cis*-elements in the *GlymaGAL* promoter.

Another possible explanation for the lack of activity of the *pGAL*-1kb sequence is the frequency and the spatial organization of its *cis*-elements. Furthermore, the promoter activity analysis of *pGAL*-1kb was carried out in a heterologous system, which may also explain the negative result. Soybean and *A. thaliana* evolutionarily split 90 million years ago, so *cis-*elements and transcription factors and promoter organization is likely to differ between these species and to interfere in the regulation of *pGAL*-1kb (Shoemaker *et al.*, 1996; Mühlhausen *et al.*, 2013).

The use of the drought stress-responsive promoter originally isolated from the soybean genome may have advantages as a biotechnological tool to improve drought stress tolerance in soybean cultivars. Its use may minimize the unexpected promoter activity caused by cryptic *cis*-elements introduced with the use of foreign DNA in soybean transgenic cultivars. In addition to *pGAL*-2kb, two other soybean promoters, *GmNCED1* and *GmMYB363*, which present activity in response to abiotic stresses, were recently characterized and patented (Li *et al.*, 2014; Zhang *et al.*, 2014).

The activity of the *GmNCED1* promoter was significantly induced by salt stress in the roots of tobacco transgenic plants (Li *et al.*, 2014). The *GmMYB363* promoter activity was induced in soybean transgenic roots under PEG 6000 treatment (Zhang *et al.*, 2014). Compared to *pGmNCED1* and *pGmMYB363*, the soybean *pGAL*-2kb promoter is active in roots as well as in leaves during drought stress. The promoter activity in both organs may represent an advantage to provide wide and effective protection under water deficit when driving the expression of drought-tolerant genes in transgenic plants. Nevertheless, *pGmMYB363* and *pGmNCED1* have shorter sequences than the *pGAL*-2kb promoter (1,384 and 1,253 bp, respectively), which is an important characteristic. However, we identified a high number of *cis*-elements in the *pGAL*-2kb promoter between 1000 and 1500 bp upstream of the start codon. Furthermore, we did not find *cis*-elements in the distal region between 1,500 and 2,000 bp. Therefore, it is important to evaluate the 1.5-kb sequence of the *pGAL* promoter to improve its use as a biotech tool. In addition, other constructs containing *pGAL*-2kb deletions may also be active as drought-responsive, as efficient as the entire sequence. Imtiaz *et al.* (2015) showed that the deletion of the 1,162-bp fragment between −2,552 (full promoter) and −1,390 significantly reduced promoter activity in leaves and roots, while no significant decrease in promoter activity was observed with further deletions from −1,390 to −780, −780 to 600 and −600 to −480. Thus, further analyses will aid in better characterizing the use of the *pGAL*-2kb promoter as a new drought stress-responsive promoter. Here we showed that the full-length *pGAL*-2kb sequence is a potential candidate for use in genetic engineering to induce a response to drought stress in soybean.

## Supplementary material

The following online material is available for this article:

Table S1 - PCR primers used in the current study.

## References

[B1] Abe H, Yamaguchi-Shinozaki K, Urao T, Iwasaki T, Hosokawa D, Shinozaki K (1997). Role of Arabidopsis MYC and MYB homologs in drought- and abscisic
acid-regulated gene expression. Plant Cell.

[B2] Abe H, Urao T, Ito T, Seki M, Shinozaki K, Yamaguchi-Shinozaki K (2003). Arabidopsis AtMYC2 (bHLH) and AtMYB2 (MYB) function as transcriptional
activators in abscisic acid signaling. Plant Cell.

[B3] Andersen CL, Jensen JL, Orntoft TF (2004). Normalization of real-time quantitative reverse transcription-PCR
data: A model-based variance estimation approach to identify genes suited for
normalization, applied to bladder and colon cancer data sets. Cancer Res.

[B4] Baker SS, Wilhelm KS, Thomashow MF (1994). The 5’-region of *Arabidopsis thaliana* cor15a has
cis-acting elements that confer cold-, drought- and ABA-regulated gene
expression. Plant Mol Biol.

[B5] Banerjee J, Sahoo DK, Dey N, Houtz RL, Maiti IB (2013). An intergenic region shared by At4g35985 and At4g35987 in
*Arabidopsis thaliana* is a tissue specific and stress inducible
bidirectional promoter analyzed in transgenic Arabidopsis and tobacco
plants. PLoS ONE.

[B6] Behnam B, Iuchi S, Fujita M, Fujita Y, Takasaki H, Osakabe Y, Yamaguchi-Shinozaki K, Kobayashi M, Shinozaki K (2013). Characterization of the promoter region of an Arabidopsis gene for
9-cis-epoxycarotenoid dioxygenase involved in dehydration-inducible
transcription. DNA Res.

[B7] Bhauso TD, Radhakrishnan T, Kumar A, Mishra GP, Dobaria JR, Patel K, Rajam MV (2014). Overexpression of bacterial mtlD gene in peanut improves drought
tolerance through accumulation of mannitol. Sci World J.

[B8] Bihmidine S, Lin J, Stone JM, Awada T, Specht JE, Clemente TE (2013). Activity of the Arabidopsis RD29A and RD29B promoter elements in
soybean under water stress. Planta.

[B9] Busk PK, Pagès M (1998). Regulation of abscisic acid-induced transcription. Plant Mol Biol.

[B10] Chan Ju L, Ha Yeon L, Woong Bom K, Bok-Sim L, Jungeun K, Raza A, Hyun AK, So Young Y, Cheol-Goo H, Suk-Yoon K (2012). Screening of tissue-specific genes and promoters in tomato by
comparing genome wide expression profiles of Arabidopsis
orthologues. Mol Cells.

[B11] Chen Y, Yordanov YS, Ma C, Strauss S, Busov VB (2013). DR5 as a reporter system to study auxin response in
Populus. Plant Cell Rep.

[B12] Clough SJ, Bent AF (1998). Floral dip: A simplified method for Agrobacterium-mediated
transformation of *Arabidopsis thaliana*. Plant J.

[B13] Conab (2012). Acompanhamento da Safra Brasileira de Grãos 2011/12. V: Sexto
Levantamento.

[B14] Czechowski T, Stitt M, Altmann T, Udvardi MK, Scheible W-R (2005). Genome-wide identification and testing of superior reference genes for
transcript normalization in Arabidopsis. Plant Physiol.

[B15] Datta K, Baisakh N, Ganguly M, Krishnan S, Yamaguchi Shinozaki K, Datta SK (2012). Overexpression of Arabidopsis and rice stress genes’ inducible
transcription factor confers drought and salinity tolerance to
rice. Plant Biotechnol J.

[B16] Doyle JJ, Doyle JL (1987). A rapid DNA isolation procedure for small quantities of fresh leaf
tissue. Phytochem Bull.

[B17] Du H, Shen X, Huang Y, Huang M, Zhang Z (2016). Overexpression of Vitreoscilla hemoglobin increases waterlogging
tolerance in Arabidopsis and maize. BMC Plant Biol.

[B18] Dubouzet JG, Sakuma Y, Ito Y, Kasuga M, Dubouzet EG, Miura S, Seki M, Shinozaki K, Yamaguchi-Shinozaki K (2003). OsDREB genes in rice, *Oryza sativa* L., encode
transcription activators that function in drought-, high-salt- and cold-responsive
gene expression. Plant J.

[B19] Engels C, Fuganti-Pagliarini R, Marin SRR, Marcelino-Guimarães FC, Oliveira MCN, Kanamori N, Mizoi J, Nakashima K, Yamaguchi-Shinozaki K, Nepomuceno AL (2013). Introduction of the rd29A: AtDREB2A CA gene into soybean
(*Glycine max* L. Merril) and its molecular characterization in
leaves and roots during dehydration. Genet Mol Biol.

[B20] Goodstein DM, Shu S, Howson R, Neupane R, Hayes RD, Fazo J, Mitros T, Dirks W, Hellsten U, Putnam N (2012). Phytozome: A comparative platform for green plant
genomics. Nucleic Acids Res.

[B21] Guimarães-Dias F, Neves-Borges AC, Viana AAB, Mesquita RO, Romano E, de Fátima Grossi-de-Sá M, Nepomuceno AL, Loureiro ME, Alves-Ferreira M (2012). Expression analysis in response to drought stress in soybean: Shedding
light on the regulation of metabolic pathway genes. Genet Mol Biol.

[B22] Hellemans J, Mortier G, De Paepe A, Speleman F, Vandesompele J (2007). qBase relative quantification framework and software for management
and automated analysis of real-time quantitative PCR data. Genome Biol.

[B23] Higo K, Ugawa Y, Iwamoto M, Korenaga T (1999). Plant cis-acting regulatory DNA elements (PLACE) database:
1999. Nucleic Acids Res.

[B24] Homrich MS, Wiebke-Strohm B, Weber RLM, Bodanese-Zanettini MH (2012). Soybean genetic transformation: A valuable tool for the functional
study of genes and the production of agronomically improved plants. Genet Mol Biol.

[B25] Hou J, Jiang P, Qi S, Zhang K, He Q, Xu C, Ding Z, Zhang K, Li K (2016). Isolation and functional validation of salinity and osmotic stress
inducible promoter from the maize type-II H+-pyrophosphatase gene by deletion
analysis in transgenic tobacco plants. PLoS ONE.

[B26] Hsieh T-H, Lee J-T, Yang P-T, Chiu L-H, Charng Y-Y, Wang Y-C, Chan M-T (2002). Heterology expression of the Arabidopsis C-repeat/dehydration response
element binding factor 1 gene confers elevated tolerance to chilling and oxidative
stresses in transgenic tomato. Plant Physiology.

[B27] Imtiaz M, Yang Y, Liu R, Xu Y, Khan MA, Wei Q, Gao J, Hong B (2015). Identification and functional characterization of the BBX24 promoter
and gene from Chrysanthemum in Arabidopsis. Plant Mol Biol.

[B28] Iwasaki T, Yamaguchi-Shinozaki K, Shinozaki K (1995). Identification of a cis-regulatory region of a gene in
*Arabidopsis thaliana* whose induction by dehydration is
mediated by abscisic acid and requires protein synthesis. Mol Gen Genet.

[B29] Jefferson RA (1989). The GUS reporter gene system. Nature.

[B30] Kasuga M, Liu Q, Miura S, Yamaguchi-Shinozaki K, Shinozaki K (1999). Improving plant drought, salt, and freezing tolerance by gene transfer
of a single stress-inducible transcription factor. Nat Biotech.

[B31] Kuma KM, Lopes-Caitar VS, Romero CCT, Silva SMH, Kuwahara MK, Carvalho MCCG, Abdelnoor RV, Dias WP, Marcelino-Guimarães FC (2015). A high efficient protocol for soybean root transformation by
*Agrobacterium rhizogenes* and most stable reference genes for
RT-qPCR analysis. Plant Cell Rep.

[B32] Lata C, Prasad M (2011). Role of DREBs in regulation of abiotic stress responses in
plants. J Exp Bot.

[B33] Li H, Fa Wei, Zhang Jie, Wang Nan, Li Qiang, Huan C (2014). Soybean adverse situation induced gene promoter and application
thereof. In Google Patents.

[B34] Luo X, Wu J, Li Y, Nan Z, Guo X, Wang Y, Zhang A, Wang Z, Xia G, Tian Y (2013). Synergistic effects of GhSOD1 and GhCAT1 overexpression in cotton
chloroplasts on enhancing tolerance to methyl viologen and salt
stresses. PLoS ONE.

[B35] Mühlhausen A, Lenser T, Mummenhoff K, Theißen G (2013). Evidence that an evolutionary transition from dehiscent to indehiscent
fruits in Lepidium (Brassicaceae) was caused by a change in the control of valve
margin identity genes. Plant J.

[B36] Murashige T, Skoog F (1962). A revised medium for a rapid growth and bioassays with tobacco tissues
cultures. Plant Physiol.

[B37] Narusaka Y, Nakashima K, Shinwari ZK, Sakuma Y, Furihata T, Abe H, Narusaka M, Shinozaki K, Yamaguchi-Shinozaki K (2003). Interaction between two cis-acting elements, ABRE and DRE, in
ABA-dependent expression of Arabidopsis rd29A gene in response to dehydration and
high-salinity stresses. The Plant Journal.

[B38] Nobres P, Patreze CM, Waltenberg FP, Correa MF, Tavano ECDR, Mendes BMJ, Alves-Ferreira M (2016). Characterization of the promoter of the homeobox gene CaHB12 in
*Coffea arabica*. Trop Plant Biol.

[B39] Novák J, Pavlu J, Novák O, Nozková-Hlavácková V, Spundová M, Hlavinka J, Koukalová S, Skalák J, Cerny M, Brzobohaty B (2013). High cytokinin levels induce a hypersensitive-like response in
tobacco. Ann Bot.

[B40] Peters S, Egert A, Stieger B, Keller F (2010). Functional identification of Arabidopsis ATSIP2 (At3g57520) as an
alkaline α-galactosidase with a substrate specificity for raffinose and an
apparent sink-specific expression pattern. Plant Cell Physiol.

[B41] Polizel AMM, Nakashima K, Yamanaka N, Farias JR, de Oliveira MC, Marin SR, Abdelnoor RV, Marcelino-Guimarães FC, Fuganti R, Rodrigues FA (2011). Molecular, anatomical and physiological properties of a genetically
modified soybean line transformed with rd29A: AtDREB1A for the improvement of
drought tolerance. Genet Mol Res.

[B42] Rahman H, Ramanathan V, Nallathambi J, Duraialagaraja S, Muthurajan R (2016). Over-expression of a NAC 67 transcription factor from finger millet
(*Eleusine coracana* L.) confers tolerance against salinity and
drought stress in rice. BMC Biotechnol.

[B43] Rippey BR (2015). The U.S. Drought of 2012. Weather and Climate Extremes.

[B44] Rodrigues FA, Marcolino-Gomes J, de Fátima Corrêa Carvalho J, do Nascimento LC, Neumaier N, Farias JRB, Carazzolle MF, Marcelino FC, Nepomuceno AL (2012). Subtractive libraries for prospecting differentially expressed genes
in the soybean under water deficit. Genet Mol Biol.

[B45] Saint Pierre C, Crossa JL, Bonnett D, Yamaguchi-Shinozaki K, Reynolds MP (2012). Phenotyping transgenic wheat for drought resistance. J Exp Bot.

[B46] Sengupta S, Mukherjee S, Basak P, Lahiri Majumder A (2015). Significance of galactinol and raffinose family oligosaccharide
synthesis in plants. Frontiers Plant Sci.

[B47] Shinozaki K, Seki M, Nanjo T (2007). Environmental stress responsive promoter. Google Patent.

[B48] Shoemaker RC, Polzin K, Labate J, Specht J, Brummer EC, Olson T, Young N, Concibido V, Wilcox J, Tamulonis JP (1996). Genome duplication in soybean (*Glycine* subgenus
*Soja*). Genetics.

[B49] Silva FAS, Azevedo CAV (2002). Versão do programa computacional Assistat para o sistema operacional
Windows. Rev Bras Prod Agroindustr.

[B50] Silvente S, Sobolev AP, Lara M (2012). Metabolite adjustments in drought tolerant and sensitive soybean
genotypes in response to water stress. PLoS ONE.

[B51] Soussi M, Ocaña A, Lluch C (1998). Effects of salt stress on growth, photosynthesis and nitrogen fixation
in chick-pea (*Cicer arietinum* L.). J Exp Bot.

[B52] Taji T, Ohsumi C, Iuchi S, Seki M, Kasuga M, Kobayashi M, Yamaguchi-Shinozaki K, Shinozaki K (2002). Important roles of drought- and cold-inducible genes for galactinol
synthase in stress tolerance in *Arabidopsis thaliana*. Plant J.

[B53] Tan B-C, Joseph LM, Deng W-T, Liu L, Li Q-B, Cline K, McCarty DR (2003). Molecular characterization of the Arabidopsis 9-cis epoxycarotenoid
dioxygenase gene family. Plant J.

[B54] Tran L-SP, Mochida K (2010). Functional genomics of soybean for improvement of productivity in
adverse conditions. Funct Integr Genomics.

[B55] Tran L-SP, Nakashima K, Sakuma Y, Simpson SD, Fujita Y, Maruyama K, Fujita M, Seki M, Shinozaki K, Yamaguchi-Shinozaki K (2004). Isolation and functional analysis of Arabidopsis stress-inducible NAC
transcription factors that bind to a drought-responsive cis-element in the
*early responsive to dehydration stress 1*
promoter. Plant Cell.

[B56] Urao T, Yamaguchi-Shinozaki K, Urao S, Shinozaki K (1993). An Arabidopsis *myb* homolog is induced by dehydration
stress and its gene product binds to the conserved MYB recognition
sequence. Plant Cell.

[B57] USDA (2016). World Agricultural Production.

[B58] Verslues PE, Agarwal M, Katiyar-Agarwal S, Zhu J, Zhu J-K (2006). Methods and concepts in quantifying resistance to drought, salt and
freezing, abiotic stresses that affect plant water status. Plant J.

[B59] Wang J-Y, Wang J-P, Yang H-F (2016). Identification and functional characterization of the NAC gene
promoter from *Populus euphratica*. Planta.

[B60] Withanage SP, Hossain MA, Kumar MS, Roslan HAB, Abdullah MP, Napis SB, Shukor NAA (2015). Overexpression of *Arabidopsis thaliana* gibberellic
acid 20 oxidase (AtGA20ox) gene enhance the vegetative growth and fiber quality in
kenaf (*Hibiscus cannabinus* L.) plants. Breed Sci.

[B61] Yamaguchi-Shinozaki K, Shinozaki K (1993). Characterization of the expression of a desiccation-responsive rd29
gene of *Arabidopsis thaliana* and analysis of its promoter in
transgenic plants. Mol Gen Genet.

[B62] Yamaguchi-Shinozaki K, Shinozaki K (1994). A novel cis-acting element in an Arabidopsis gene is involved in
responsiveness to drought, low-temperature, or high-salt stress. The Plant Cell.

[B63] Yan H, Ma L, Wang Z, Lin Z, Su J, Lu B-R (2015). Multiple tissue-specific expression of rice seed-shattering gene SH4
regulated by its promoter pSH4. Rice.

[B64] Zhang J, Chen S, Li Q, Ma B, Liu Y, Zhang W, Lin Q, He S (2014). Soybean derived drought induced type promoter GmMYB363P and
application thereof. Google patent.

[B65] Zhao S, Fernald RD (2005). Comprehensive algorithm for quantitative real-time polymerase chain
reaction. J Comp Biol.

[B66] Zulauf C (2012). Drought: Yield Loss, Revenue Loss, and Harvest Price Option.
Farmadocdaily.

