# Supplementary material for: Isolation and characterization of a promoter responsive to salt, osmotic and dehydration stresses in soybean
Source: Genet Mol Biol. 2017 Mar 27;40(1 Suppl 1):226–37. doi: 10.1590/1678-4685-GMB-2016-0052 (PMC5452143; doi:10.1590/1678-4685-GMB-2016-0052)
Supplement: Supplementary file 1 [file 1415-4757-gmb-1678-4685-GMB-2016-0052-Suppl01.pdf]

Table S1 - PCR primers used in the current study.

Primer	Forward 5`-3`	Reverse 5`-3`	Tm ºC
**pGAL-1kb**	CACCCTAATCAAGCGTTGTT	TTTCGAACACTTCACCACTG	55
**pGAL-2kb**	CACCTAGTTATTTGACTGGATTC		55
35S	CACCACTAGAGCCAAGCTGATCT	TCGACTAGAATAGTAAATTGTAATGTT	55
**M13**	CAGGAAACAGCTATGAC	GTAAAACGACGGCCAG	52
**At4g34270**	GTGAAAACTGTTGGAGAGAAGCAA	TCAACTGGATACCCTTTCGCA	60
**At4g38070**	GAAAGCAAAGGCGGTGAGAG	CAAGGCACACTTGGTTCTTCC	60
EGFP	TGGTGCCCATCCTGGTCGAGC	GCTCGATGCGGTTCACCAGGG	60
uidA	TGATAGCGCGTGACAAAAA	CGAAATATTCCCGTGCACTT	60
